# The Healthcare Sector Employer’s Duty of Care: Implications for Worker Well-Being

**DOI:** 10.3390/ijerph18116015

**Published:** 2021-06-03

**Authors:** Melissa McDiarmid, Marian Condon, Joanna Gaitens

**Affiliations:** School of Medicine, University of Maryland, Baltimore, MD 21201, USA; mmcdiarm@som.umaryland.edu (M.M.); jgaitens@som.umaryland.edu (J.G.)

**Keywords:** well-being, burnout, COVID, healthcare worker, duty of care

## Abstract

Pandemic diseases of this century have differentially targeted healthcare workers globally. These infections include Severe Acute Respiratory Syndrome SARS, the Middle East respiratory syndrome coronavirus Middle East respiratory syndrome coronavirus (MERS-CoV) and Ebola. The COVID-19 pandemic has continued this pattern, putting healthcare workers at extreme risk. Just as healthcare workers have historically been committed to the service of their patients, providing needed care, termed their “duty of care”, so too do healthcare employers have a similar ethical duty to provide care toward their employees arising from historical common law requirements. This paper reports on results of a narrative review performed to assess COVID-19 exposure and disease development in healthcare workers as a function of employer duty of care program elements adopted in the workplace. Significant duty of care deficiencies reported early in the pandemic most commonly involved lack of personal protective equipment (PPE) availability. Beyond worker safety, we also provide evidence that an additional benefit of employer duty of care actions is a greater sense of employee well-being, thus aiding in the prevention of healthcare worker burnout.

## 1. Introduction

Respiratory infectious diseases of this century, beginning with the severe acute respiratory syndrome (SARS) outbreak of 2003, have differentially targeted healthcare workers globally. Among the 8000 affected with SARS in 29 countries, more than twenty percent were healthcare workers [1]. This included both clinically assigned workers as well as those providing non-clinical support functions [2].

Likewise, the Middle East respiratory syndrome coronavirus (MERS-CoV) emergence in 2012 was associated with high numbers of healthcare worker infections with many cases associated with ‘super-spreader’ events [3]. Now numbering more than 2400 cases, an additional 219 were reported by the World Health Organization (WHO) in 2019, of which 52 were linked to hospital transmission, with half of those being infections in healthcare workers [4].

Prior to these emerging respiratory infections, the importance of nosocomial transmission of tuberculosis and its threat to worker safety was well known [5]. Most recently, the bitter lessons of Ebola, which also targeted healthcare workers differentially, at more than a ten-fold higher rate than community members [6], suggested early in the COVID-19 outbreak that the healthcare workforce was likely at extreme risk. This awareness warrants an examination of worker protections in place to prevent exposure and minimize harm to workers’ health and safety.

Although statute-specific legal requirements of employers toward their workers’ safety have only been instituted in their present form in well-resourced countries over the past 50 years [7,8], the broader employer responsibility toward providing “reasonable care” toward the workforce existed much earlier under common law requirements [9]. Specific expectations of employers here include the prevention of illness, injury and death of workers, providing safe systems of work, safety equipment and generally assuring the health, safety and welfare of workers. These previously existing common law responsibilities have been formalized and acknowledged and are now being enforced under specific, legally binding statues in many countries globally.

The British Health and Safety at Work Act of 1974 specified in a provision titled: General duties of employers to their employees, that “It shall be the duty of every employer to secure, so far as is reasonably practicable, the health, safety and welfare at work of all his employees” [8]. In this legal framework, the employer is the ‘duty holder’ of this safety and health responsibility. While this duty is somewhat broad, the general approach to worker safety and health protections depends on performing risk assessments for hazards and establishing policies and procedures to minimize harm [10]. There are also specific provisions for different economic sectors.

The International Labor Office (ILO) Occupational Safety and Health Convention (C155) of 1981 had earlier challenged member states, in collaboration with employer and worker representatives, to “formulate, implement and periodically review a coherent national policy on occupational safety, occupational health and the working environment” [11]. Indeed, the British Health and Safety at Work Act, described above, took its language from the ILO Convention which states “It shall be the duty of every employer to ensure, so far as is reasonably practicable, the health, safety and welfare at work of all his employees”. It further states that employers must ensure preventive measures are taken for the protection of life, and physical and mental health of workers. This convention and a related 2002 protocol have been ratified by 69 countries [12].

The ethical and legal responsibilities to protect the health and safety of employees are also binding in the healthcare sector and even in emergency settings with evolving risk scenarios. The employer’s duty has come to the fore in all the recent respiratory public health emergencies described above.

The employer’s duty mirrors that of healthcare workers, who have historically, in professional ethics codes, committed to the service of their patients, providing protections from risk of harm, termed one’s “duty of care” [13]. This has also been expected during pandemics and other emergencies [14]. In the SARS outbreak of 2003, with the heavy toll of illness and death among healthcare workers, some authors suggested a reciprocal obligation of employers toward their employees to make their added risk-taking as safe as possible [15,16]. This could be achieved, it was proposed, through worker training, performing risk assessments and providing the personal protective equipment (PPE) workers needed. All these actions are the elements of the common law and formal statutes describing the employer’s ‘duty’ to provide for the health and safety of their employees.

Ethical responsibilities of the employer under their duty of care include clearly communicating expectations to staff during a pandemic and providing psychosocial support, sufficient skills training and required resources, especially PPE. Additional prevention services, such as vaccines when available and medical care if illness results, should also be provided [17].

The WHO/ILO document “Occupational Safety and Health in Public Health Emergencies” lays out similar employer responsibilities, which include providing:

- Safe working conditions (performing risk assessment and management planning) and
- Information for workers (skills training regarding PPE).

In addition, employers are bound to report occupational illness and injury statistics to government agencies. Worker responsibilities include participating in training, following OSH (occupational safety and health) guidelines and reporting hazards to the employer [18]. Of interest, workers also have the right to refuse unsafe work.

WHO also has COVID-specific resources that add detail to the above general approaches to employer and worker responsibilities [19]. These include calling for “healthy, safe and decent” working conditions for workers caring for COVID-19 patients [20] and the basic elements of a comprehensive safety program including infection control (IC) and other safe work practices (PPE use and psychosocial support).

Ideally, there should be a balance between employer and healthcare worker duty of care to ensure optimal patient safety and worker protections. The objectives of this paper are to describe the effect of employer duty of care and occupational safety and health (OSH) responsibilities on COVID-19 disease outcomes in the healthcare workforce. Repeating themes identifying the presence of specific duty of care domains of responsibility or deficiencies in these domains will be reported where possible, related to healthcare worker outcomes. Finally, the synergies between a robust duty of care culture and worker well-being will also discussed.

## 2. Methods

A narrative review was performed to assess COVID-19 exposure and disease development in healthcare workers as a function of employer duty of care program elements deployed in the workplace. Peer-reviewed articles as well as public news sources were examined using search terms including “employer duty of care in COVID-19” or “duty of care in the COVID pandemic”, in both PubMed and Google search engines. These articles and sources were culled to address only health sector employers including acute, long-term care and home care settings. Specific duty of care elements provided by, or which failed to be provided by the employer were identified. Search dates included January 2020 through January 2021. An additional query was also performed in PubMed to examine the literature by identifying “risk factors for COVID development in healthcare workers” with various spellings of COVID or COVID-19 included.

## 3. Results

The narrative review was restricted to publications from January 2020 through January 2021, and thus primarily illustrates the first wave of the pandemic crisis and its immediate aftermath through to the early rise of the second wave in the fall of 2020. This review required two different search queries to obtain sufficient output to assess employer duty of care in the context of healthcare worker disease outcome.

The first query specifically used the term ‘duty of care’ and yielded 128 papers, of which only ten were specific to the healthcare sector and sufficiently detailed to contribute examples. The second queried for ‘risk factors for health worker COVID infection’. This resulted in 46 citations, of which 12 were contributory to assessing facility-based employer duty of care safety program elements, which could then be aligned with duty of care domains. All of these papers were from the peer-reviewed literature.

Two additional non-peer-reviewed references included a longitudinal report by a health news service on the COVID crisis in the healthcare workforce, largely covering the US and UK experience, and a serially updated report from a global non-governmental organization (NGO) covering the same topic internationally.

The narrative literature review query using the ‘risk factors’ term yielded a richer description of working conditions and duty of care domains present in healthcare facilities than did the query using the term ‘duty of care’. The latter query yielded more general discussions of employer legal or ethical requirements.

### 3.1. Risk Factors for Healthcare Worker COVID-19 Infection and Reported Safety Deficiencies

Early in the pandemic, comprehensive reporting of healthcare worker COVID-19 illness and death rates was not easily available. It was not until September 2020, six months after the onset of the pandemic, that the WHO reported that healthcare workers represented 14% of the COVID-19 disease burden globally [21,22]. This was in line with the experience of hard-hit countries during the outbreak’s first wave, such as Spain, Italy and the US The US reported that 18% of cases occurred in healthcare workers early in the pandemic [23]. Additionally, early on, between 28 February 2020, and 23 April 2020, Spain reported that 20.4% of its cases had occurred in healthcare workers, and during the same time period Italy reported a 10.7% rate of infection among healthcare workers [24,25].

To assess comparative risk to the healthcare workforce, a prospective, observational cohort study in the UK and the US enrolled healthcare workers and the general community during a thirty-day period between late March and late April 2020, using the COVID-19 Symptom Study smartphone application. The investigators found that healthcare workers were at an increased risk of reporting a positive COVID-19 test of at least 3 fold compared to community reporting (adjusted HR = 3.4, 95% CI 3.37–3.43) [26]. The authors noted that the excess was especially high among Black, Asian and minority ethnic healthcare workers and in workers reporting direct patient contact, lack of PPE, or in those who were required to reuse PPE. Although this study was based on self-report, the authors noted that any misclassification bias would have been non-differential and that the findings were sufficiently robust in subsequent sensitivity and secondary analyses.

In a study from the Minnesota Department of Health of over 21,000 COVID-19 exposures in 17,000 healthcare workers reported to a state surveillance program, 66% involved patient care events and 34% involved non-patient events, although much of this latter group included co-workers exposed by an infectious colleague at work [27]. For higher-risk exposures, including aerosol-generating procedures and proximity or longer exposure durations to a positive person, PPE was statistically less likely to have been used by workers in long-term care or congregant settings, as opposed to acute care settings. Mask use for source control by positive patients or residents was also less likely in long-term care and congregant settings. Staff was also more likely to work while experiencing symptoms when in the long-term care setting [27]. Note was made of some deviations from state guidance in risk assessments of exposure events performed in some facilities (an administrative policy or training issue). Limits in infection prevention capacity and training in long-term care, which has been reported elsewhere in these settings, were also mentioned [28].

In September of 2020, using multiple sources (memorial pages, government figures, lists compiled by national medical associations, and lists and obituaries published in media around the world), Amnesty International, through their international network, reported that over 7000 healthcare workers had died from COVID-19. As of that date, they reported at least 1320 healthcare worker deaths in Mexico, the highest among countries at the time, followed by the US (1077) and Brazil (643). Also listed were concerning numbers from South Africa (240) and India (573), reflecting recent raised infection rates in those countries. The agency compared this September 2020 figure to their previously reported 3000 healthcare worker deaths through mid-July [29].

This account also highlighted many duty of care deficiencies found in COVID-19 death investigations, with workers raising safety concerns. These included inadequate provision of PPE and lack of psychosocial support. They also noted that contracted workers were at increased risk due to less protection observed through employment outsourcing, which may blur lines of which employer has ‘duty holder’ responsibility [29].

In a systematic review by Gholani and colleagues [30], risk factors for COVID-19 infection in healthcare workers included the lack and re-use of PPE, but also other work practice deficiencies, including sub-optimal hand hygiene by workers (a training issue), failure to place a mask on a suspect patient and workers not wearing masks even when provided. Also associated with healthcare worker COVID-19 disease was workplace setting (locations where aerosol procedures occur and areas where intensity of exposure to COVID-19 patients is high), including nursing homes [26].

The Kaiser Health Network, which has followed this story since the beginning of the pandemic, together with The Guardian, reported in October 2020 the deaths of 2900 US healthcare workers. These included both acute care, nursing home (long-term care) and home care workers. Sources of the report were a review of governmental and public data sources, labor union websites, posted obituaries, as well as required reporting to the government of worker illness in long-term care and home care. In more than 300 interviews with family and others who knew the deceased, concerns about lack of PPE were identified in a third of the cases [31].

While the major focus on healthcare worker COVID-19 illness has been on acute care facilities, as the Kaiser report reflects, other environments also threaten the health of workers providing both health and personal care, principally in long-term care (LTC) and home care settings. Surprisingly, while the tragedy of COVID-19 illness and death among elders in LTC has been reported globally, in the US, workers account for close to half (46%) of COVID-19 nursing home infections but only about 10% of deaths according to the US Centers for Medicare and Medicaid Services [32].

The high-risk environment in LTC has been linked to several factors, including lack of access to testing early in the pandemic and contact tracing constraints, but also to more long-standing issues of staffing and PPE shortages. Chronic staffing shortages have led to the use of healthcare personnel from staffing agencies, or shared staff between co-owned facilities, permitting infection to be shared. Additionally, PPE shortages are common and recurring. A recent study from a national COVID-19 nursing home database of over 15,000 facilities found about 20% reported severe PPE shortages during the pandemic, with a similar share reporting significant staff shortages in nursing and other staff [33]. The authors point out that both these types of shortages threaten the health of residents and staff alike.

One could also argue that such shortages are preventable and suggest a duty of care deficiency. In the US, both Centers for Disease Control and Prevention (CDC) and other governmental organizations have issued guidance to assist in the prevention and management of outbreaks among LTC residents and staff [34]. Many of these recommendations were not innovative but based on long-standing IC practices and reasonable employment strategies, such as providing sick leave, so ill workers could stay home when sick [35]. These examples suggest structural deficiencies that could be remedied to prevent COVID-19 spread.

While the LTC worker is far from the relatively more robust safety structures of fixed-site, acute care organizations, the home care worker is even farther away. These healthcare workers provide both personal care, such as bathing and dressing, and medical care, such as taking vital signs and providing wound care, to community-living clients who require assistance [36]. The unique risks to this group include caring for multiple clients as well as their longer duration of ‘exposure’ to the client given their job duties of personal care, compared to acute care. While some authors use the term ‘vulnerable’ for these workers [37], indeed their working conditions are closer to precarious work, that is, non-standard, insecure, unprotected and outside a typical employment relationship [38], when factoring in other social determinants of health commonly encountered in this setting. These include low-wage workers of color, generally working for small companies and providing care for home-bound frail elderly and sick clients. It must be noted, however, that during the pandemic, these workers were the protective bulwark of hard-hit areas in the US, such as New York and New Jersey, keeping their clients out of already critically crowded emergency departments in hospitals. Nevertheless, in a survey of 33 home health workers, a familiar pattern of PPE shortage was found, as well as sometimes inadequate training [36].

### 3.2. Repeating Themes in Duty of Care Failures Reported in the Narrative Review

A summary of duty of care deficiencies reported in healthcare worker COVID-19 exposure and infection studies derived from the narrative review, including those discussed above, are displayed in Table 1. These reports are illustrative of five repeating themes recounted in the literature that threatened worker health and safety.

The first major heading in the table discusses the employer’s responsibility to plan for safe work. This was described in the introduction as a key responsibility of the safety duty holder. As seen in the table, there was a widespread lack of planning even for IC protocols which should have been a more well-recognized employer responsibility given the ubiquity of IC activities even in conventional healthcare delivery.

The overwhelming deficiency reported was the failure to provide material resources for patient care and worker safety, specifically the lack of PPE. This led to unsafe re-use or use of lesser forms of protection. Another major deficiency commonly reported was the lack of training for both safe PPE use and infection prevention. Examples include failure to train on masking a source patient and on optimal hand hygiene.

Additionally, the ability to provide adequate staffing and manageable workloads, which has been repeatedly raised as a determinant of positive working conditions in the context of conventional care, was even more challenged during the COVID patient surge. Finally, related to positive working conditions was the workers’ perception of psychosocial support received from the organization. Each of these five broad areas of responsibilities, displayed in the table, are basic expectations of employer duty of care. Each of these five broad areas of responsibilities, displayed in the table, are basic expectations of employer duty of care, and were repeatedly cited as lacking in the articles reviewed.

## 4. Discussion

### 4.1. Benefits of an Employer’s Duty of Care Commitment

The basic elements of an employer’s duty of care toward the healthcare workforce are certainly present in their IC and broader safety obligations and so are not unknown to the healthcare sector employer. However, often the focus of such IC and safety provisions patient safety driven and does not encompass the safety of the workforce, the larger organization or the system [63]. Moreover, such safety programs typically address these obligations during conventional rather than contingency or crisis levels of care. Even though much has been written recently about emergency preparedness generally and pandemic readiness specifically, resource constraints compete with investments in harm avoidance, the benefits of which are in the future and perhaps too vague to appreciate.

One recent example of the benefits of an employer’s commitment to duty of care taken from the recent Ebola outbreak of 2014–2015 may prove instructive here. The largely WHO-led response to the outbreak in West Africa resulted in approximately 815 worker deaths, and a disease incidence rate of 30–44 per 1000 healthcare worker responders [6]. This is compared to the Ebola incidence rate of Médecins Sans Frontiéres (MSF) healthcare workers of about 4.3 per 1000 [64].

A significant difference between the two groups’ experience was MSF’s agency-wide commitment toward employer duty of care. Operationalized through new site risk assessments and structured policies beyond IC, MSF attempts to prevent exposure through safety and emergency procedures design, training and risk communication with workers and with follow-up of worker injury and illness [65]. This commitment to safety likely contributed to the almost ten-fold lower worker incidence rate in the MSF teams compared to those of WHO. The elements of the MSF program include duty of care guidelines and legislation internationally accepted.

Thus, beyond a high-minded ethical notion of justice, the employer’s duty of care can demonstrate tangible results in the form of life-saving differences in infection and death rates. This protects not only the healthcare work force but a facility or healthcare system’s response mission during pandemic emergencies.

### 4.2. Navigating from Prevention of Worker Harm toward Worker Well-Being

While the employer’s duty of care focus is on preventing foreseeable risk of harm, beyond mere harm avoidance, another benefit of a duty of care disposition arises from the healthcare worker burnout prevention experience.

The poor state of healthcare workers’ mental health has been widely reported in the literature and the popular press recently with much of it attributed to worker burnout [66,67]. Burnout here is defined as a state of exhaustion, cynicism and inefficiency driven by factors such as workload and job demands, resource constraints, and misalignment of organizational culture and values [68]. Burnout has also been linked to physician turnover, and to declines in care quality and patient safety [67,69].

Burnout is not new and has been widely reported, long before the pandemic [70], with many of its drivers being attributed to raised expectations of healthcare worker productivity, efforts limiting costs of care and excessive documentation requirements of the electronic health record. As many current reports illustrate, these same factors are evident and amplified in the COVID-19 pandemic, with the surge of patient demands for care in unprepared healthcare systems.

Using a questionnaire to explore the core burnout domain of emotional exhaustion, a cross-sectional study of healthcare workers caring for COVID-19 patients found that about half of 2700 participants from 60 countries reported burnout. Factors associated with burnout included ‘work impacting household activities’ (time pressures), feeling pushed beyond training, exposure to COVID-19 patients and making ‘life-or-death’ decisions. One factor found to be protective against burnout was adequate PPE [42]. In a British study, symptoms of moderate to severe burnout were reported more frequently by healthcare worker participants if they were younger, female, redeployed from a usual assignment (possibly outside their scope of training or familiarity) and if working on a COVID-19 unit [55].

Examples of factors driving burnout in COVID-19 align broadly with domains of burnout risk in general, including organizational culture and values misalignment; workload and job demands; and lack of control, inefficiency and resource constraints. Many of these domains are employer duty of care responsibilities.

The similarities in origins of burnout suggest there may be shared solutions from the burnout prevention literature applicable to the COVID-19 context. First, burnout is increasingly recognized not as a diagnosis of the individual worker, but of a work organization [71]. Hence, if it derives from the way work is organized, then the solution must come from the organization.

Actions by organizational leadership to address burnout include realigning organizational culture and values and promoting supportive communities at work [68]. Specifically, this must include organizational adjustment of productivity expectations; use of support staff to maximize clinician efficiency; an examination and commitment to culture, values, safety and equity; and enhanced flexibility of schedules. These same approaches contribute significantly toward meeting an employer’s duty of care. Organizational management and psycho-social support of staff and the visibility and accessibility of leadership were repeatedly reported as promoting staff well-being in the COVID context [45,47,52,56,57].

The most visible evidence of an employer’s active support of staff physical well-being during COVID-19 was providing availability of adequate PPE [72]. For example, Morgantini reported that the provision of PPE was protective against burnout in her large study of COVID responders [51]. However, Table 1 shows that the most commonly reported and concrete employer duty of care failure was lack of PPE.

Not only is providing PPE a highly visible demonstration of an employer’s organizational commitment to safety [63,73], but it has also been found to be an influential determinant of safety culture confidence and of healthcare workers’ willingness to report for duty in a pandemic [73]. However, the mere availability of PPE is not sufficient protection without adequate training on its use.

## 5. Conclusions

Addressing the healthcare sector employer’s duty of care obligations presents challenges, even in conventional circumstances of healthcare operation, requiring focused effort on safe work policies, providing material resources for patient care and staff protection, on-going safety training, adequate staffing, pay and benefits and attention to staff psycho-social needs. Investing in such efforts, however, provides significant benefits realized not only with respect to staff safety improvements but also in worker well-being, which assists in preventing burnout. Such a duty of care approach establishes an invaluable foundation upon which to build critical response functions during public health emergencies, preserving the healthcare system’s infrastructure and mission while sustaining resiliency in the healthcare workforce.

## Figures and Tables

**Table 1:** Selected examples of duty of care failures in COVID protections.

Employer Responsibilities	Specific Actions	COVID Duty of Care Failure	References
Plan for Safe Work (protocol and policy development)	Anticipate and plan for safe work	Lack of funding for pandemic preparedness	[39,40]
	Involve workers in Occupational Health and Safety (OHS) Committees	Lack of worker participation in OHS Committees	[29]
	Establish infection control (IC) protocols	Lack of clear IC protocols	[27,28,29,41,42,43]
Provide Material Resources for Safe Patient Care and Worker Protection	Provide personal protective equipment (PPE)	Lack of PPE; global supply chain disruption due to non-availability (poor planning) resulting in inappropriate use, reuse, or failure to provide required protections	[26,27,29,30,31,33,36,42,43,44,45,46,47,48,49,50]
	Provide materials needed for patient care	Lack of drugs, ventilators, intensive care unit beds	[44]
Provide Safety Training	Ensure adequate safety training	Inadequate safety training (safe use of PPE, work practices)	[27,28,30,36,51]
	Provide IC training	Failure to train in IC (e.g., lack of source patient masking)	[45,52]
	Provide clear communication	Lack of information, changing information (e.g., changing mask use guidance)	[43,44,50]
Address Safe Staffing and Fair Pay	Ensure adequate staffing and manageable workloads	Lack of staffing/excessive workload	[42,44]
		Duration of work shifts/number of patients/Prolonged contact with COVID patient/Lack of rest	[26,30,42,48,49,53,54]
		Ward assignment to complex patients; beyond scope of training	[51,55]
		Precarious contracts for emergency recruits	[29]
	Provide adequate pay and benefits (i.e., sick leave)	Inadequate pay or lack of hazard pay (especially in developing countries) for healthcare support and direct care workers	[29,56]
		Staff working with while experiencing COVID symptoms	[27]
Provide Psycho-social Support	Offer mental health/psychosocial support	Lack of mental health/psychosocial support	[29,44,50,51,52,55,57,58]
	Protect health workers from violence/harassment	Public feared contact with health workers as source of exposure	[59,60,61,62]

## Data Availability

No new data were created or analyzed in this study. Data sharing is not applicable to this article.
